# Extracellular Vesicles Carry HIV Env and Facilitate Hiv Infection of Human Lymphoid Tissue

**DOI:** 10.1038/s41598-017-01739-8

**Published:** 2017-05-10

**Authors:** Anush Arakelyan, Wendy Fitzgerald, Sonia Zicari, Christophe Vanpouille, Leonid Margolis

**Affiliations:** Section of Intercellular Interactions, Eunice-Kennedy National Institute of Child Health and Human Development, National Institutes of Health, Bethesda, MD USA

## Abstract

Cells productively infected with HIV-1 release virions along with extracellular vesicles (EVs) whose biogenesis, size, and physical properties resemble those of retroviruses. Here, we found that a significant number of EVs (exosomes) released by HIV-1 infected cells carry gp120 (Env), a viral protein that mediates virus attachment and fusion to target cells, and also facilitates HIV infection in various indirect ways. Depletion of viral preparations of EVs, in particular of those that carry gp120, decreases viral infection of human lymphoid tissue *ex vivo*. Thus, EVs that carry Env identified in our work seem to facilitate HIV infection and therefore may constitute a new therapeutic target for antiviral strategy.

## Introduction

It is well established that various cells *in vivo* and *in vitro* release extracellular vesicles (EVs) of various size and biogenesis^1^. Many of these vesicles (exosomes) are of the same size as retroviruses, in particular HIV, and are generated inside the cells along the pathways similar to these viruses^2, 3^. Also, these EVs may incorporate proteins that are common to viruses (e.g., tetraspanins) as well as viral genetic material^4, 5^.

Until recently EVs were considered to be “cell dust” but now EVs, in particular the small ones (less than 300 nm), are widely studied as a system of cell-cell communication that changes the status of the cells they interact with^6, 7^. EVs seem to affect viral infection^8–12^, although, the data on the actual effects of EVs on viral infection are controversial and the mechanisms of these effects remain to be investigated.

Analysis of EVs generated by infected cells as well as the effects of EVs on viral infection are complicated by the fact that it is almost impossible to separate them from virions in particular from HIV because of the similarities in size and physical properties. Therefore, any HIV preparation is in fact a mixture of HIV virions and EVs.

Here, we overcame some of these problems by segregating EVs through CD45 and/or acetylcholinesterase (AChE), two proteins that are not incorporated into HIV membranes^13–15^ and thus can be used to distinguish EVs from HIV virions. Using our nanotechnology “flow virometry”^16^, we found that a significant number of EVs generated in HIV-infected cells carry HIV Env, thus being indistinguishable from “defective” viruses. These EVs facilitate viral infection in human lymphoid tissue *ex vivo*, a system that reflects many aspects of HIV infection of lymphoid tissue *in vivo* where the critical events of HIV pathogenesis occur^17, 18^.

## Results

### EVs released by HIV-infected cells carry Env

**Figure 1:**
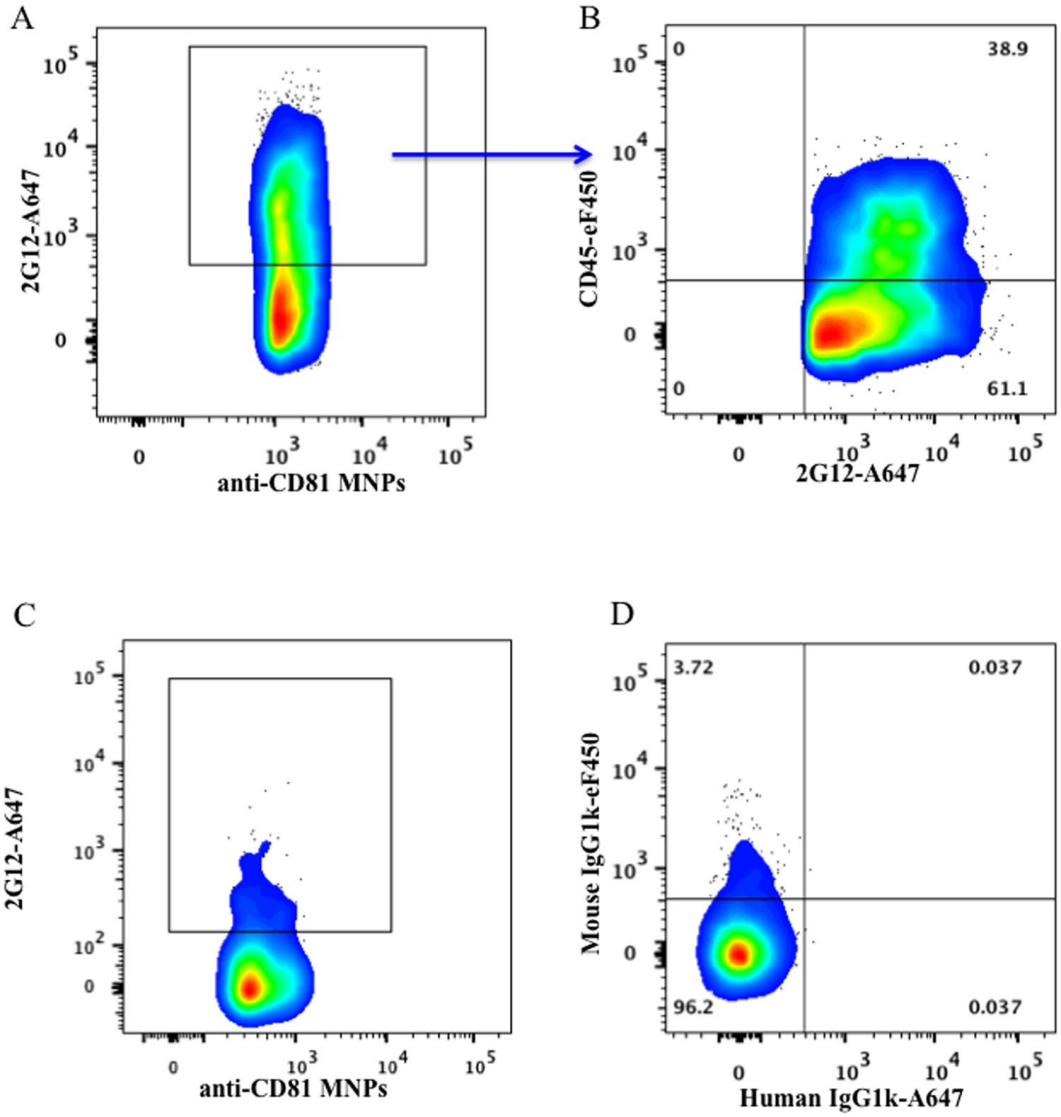
Analysis of EVs and HIV-1 captured with CD81 MNPs. A preparation of HIV_SF162_ (containing both viruses and EVs) was incubated with CD81 MNPs and the captured particles were stained for CD45 with anti-CD45-eFluor 450 and for gp120 with 2G12-Alexa Fluor 647 antibodies. (**A**) Particles (EVs and/or virions) captured with CD81 MNPs carrying gp120. (**B**) EVs (CD45+) captured with CD81 MNPs carrying gp120. (**C**) 2G12 specificity control: EVs released by uninfected PBMCs captured with CD81 MNPs negative for 2G12-Alexa Fuor 647. (**D**) Isotype control: Staining with mouse IgG1k-eFluor 450 and human IgG1k-Alexa Fluor 647 antibodies. Presented are data from one representative experiment out of five.

In these experiments we distinguished EVs from HIV virions by the presence of either CD45 or AChE, which are not incorporated into virions in the course of their biogenesis^13–15^. We used CD81, a tetraspanin that is incorporated in both HIV and EVs^4^, to fish out both entities for further analysis of their surface antigens. We added magnetic nanoparticles (MNPs) coupled to anti-CD81 antibodies to the preparation of HIV_SF162_, a prototypical CCR5-tropic HIV produced by PBMC. Then, we stained the preparation for gp120 with 2G12 fluorescently labeled antibodies, which bind to clusters of high mannose–type glycans on the outer domain of gp120^19, 20^ recognizing both monomeric and trimeric Envs, and for CD45 using specific fluorescently labeled antibodies. The results of these experiments are presented in Fig. 1. Almost all HIV (CD45 negative) particles, as expected, carry gp120. However, unexpectedly, a significant fraction of events (on average 52.6 ± 5.7% (n = 5)) positive for gp120 carry CD45, thus presumably representing EVs (Fig. 1B). As a control for specificity of gp120 staining, we used EVs released by uninfected PBMC (Fig. 1C).

**Figure 2:**
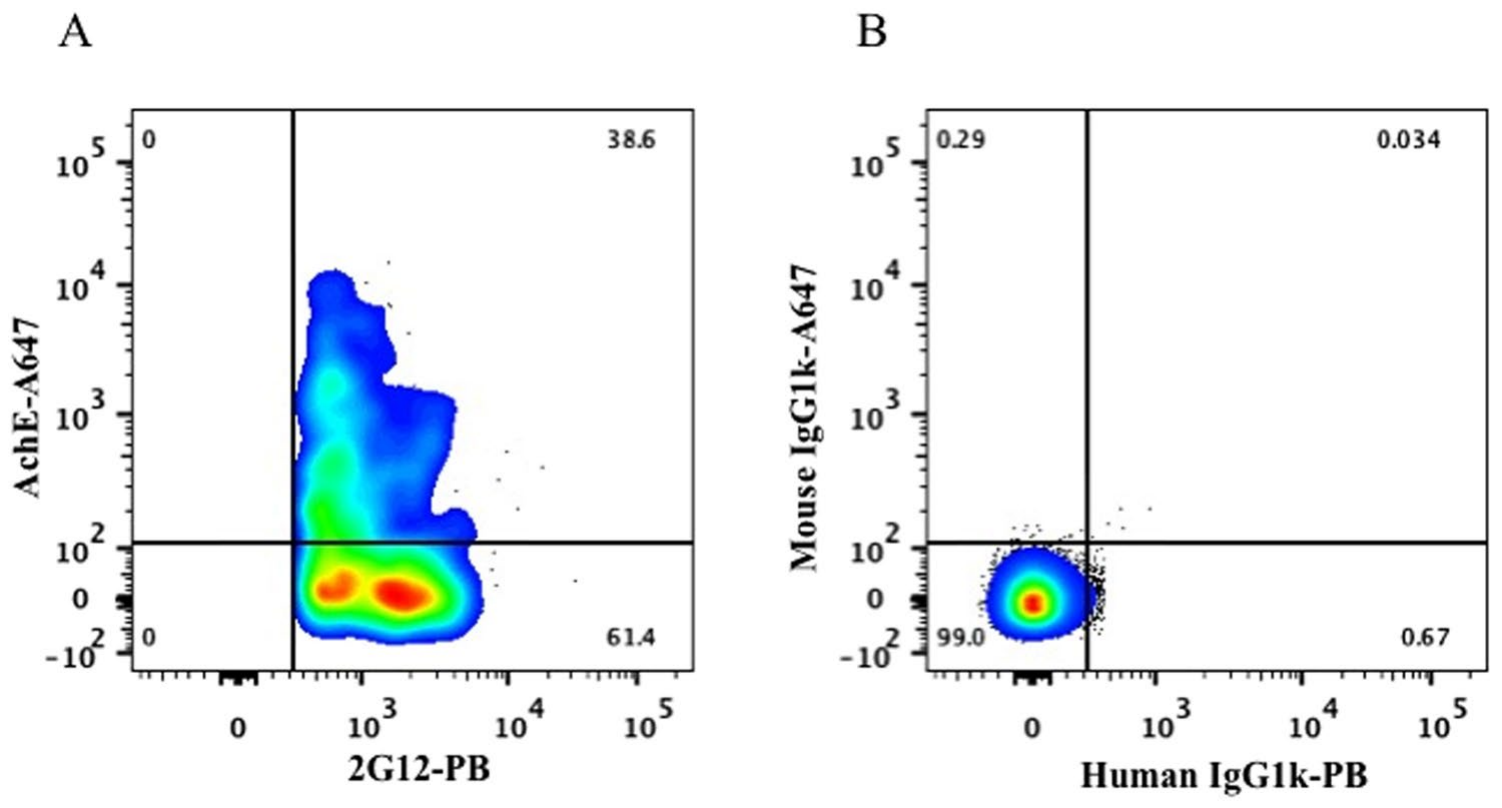
Analysis of EVs expressing gp120 in HIV_SF162_ preparation. (**A**) A preparation of HIV_SF162_ (containing both viruses and EVs) was incubated with CD81-MNPs and the captured particles were stained for AChE with anti-AChE-Alexa Fluor 647 and for gp120 with 2G12-Pacific Blue antibodies. (**B**) Isotype control: Staining with mouse IgG1k-Alexa Fluor 647 and human IgG1k-Pacific Blue. Presented are data from one representative experiment out of three.

**Figure 3:**
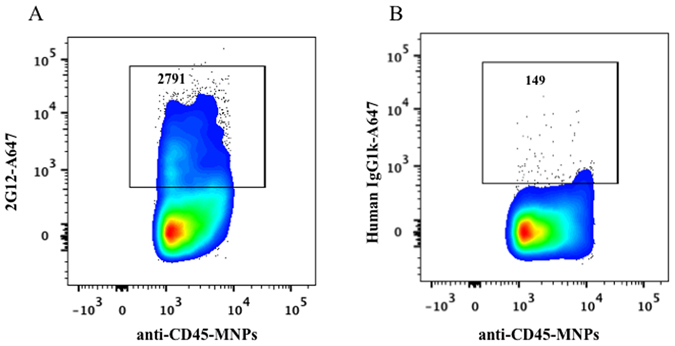
Analysis of EVs captured by CD45 MNPs in HIV_SF162_ preparation. (**A**) A preparation of HIV_SF162_ (containing both viruses and EVs) was incubated with CD45-MNPs and the captured particles were stained for gp120 with 2G12-Alexa Fluor 647 antibodies. (**B**) Isotype control: Staining with human IgG1k-Alexa Fluor 647. Indicated are the numbers of acquired events from both preparations.

The presence of gp120 in the EV membrane was confirmed in the next series of experiments in which we discriminated EVs from HIV virions by the presence of AChE^13, 15^. On average 40 ± 0.6% (n = 3) of the particles captured through CD81 and being positive for 2G12 were identified as EVs since they carried AChE (Fig. 2). The existence of gp120-positive EVs was further substantiated in experiments in which we captured EVs with anti-CD45-MNPs and stained with 2G12 antibodies (Fig. 3).

**Figure 4:**
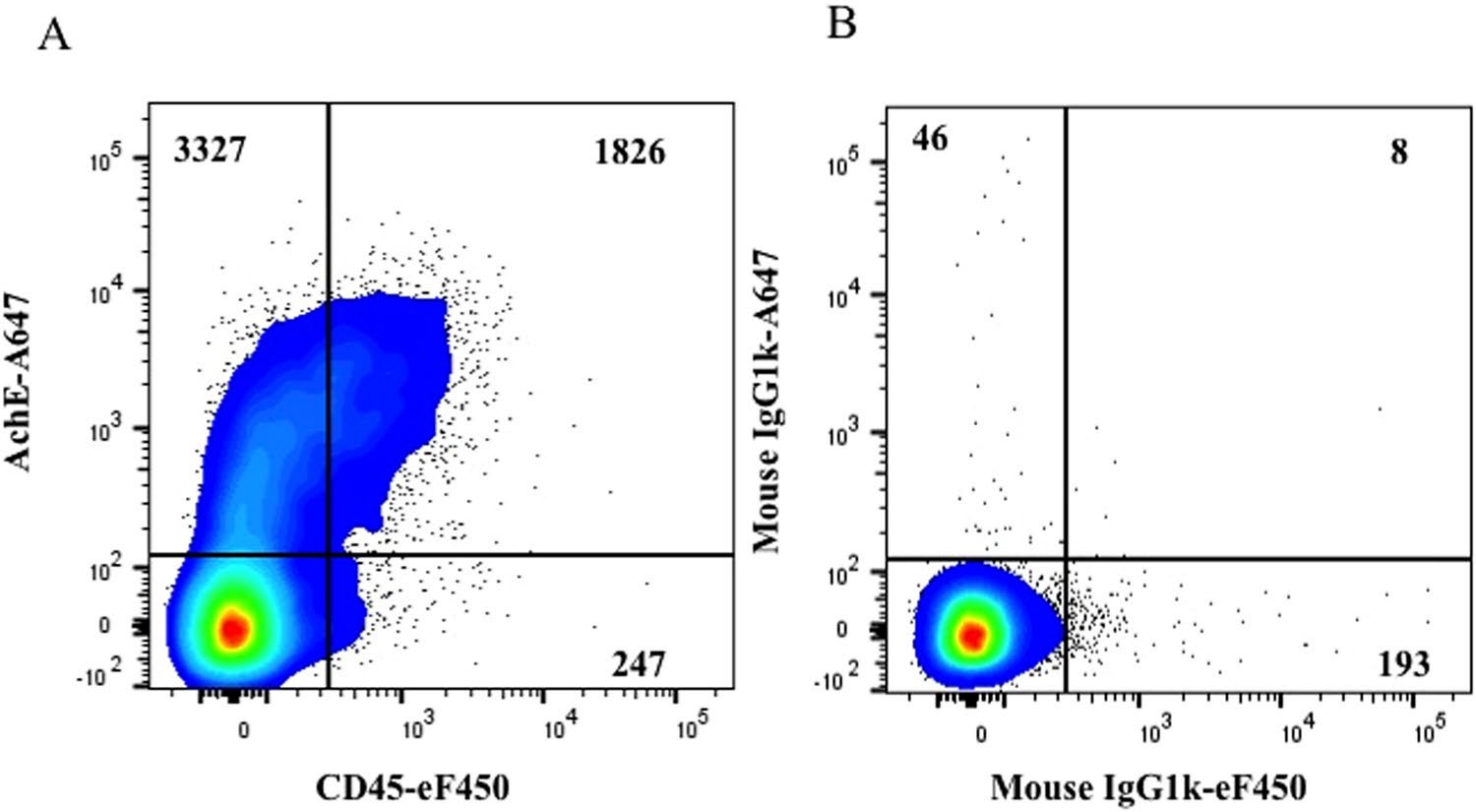
Analysis of EVs captured by PG16-MNPs. (**A**) A preparation of HIV_SF162_ (containing both viruses and EVs) was incubated with PG16-MNPs and the captured particles were stained with anti-AChE-Alexa Fluor 647 and anti-CD45-eF450 antibodies. (**B**) Isotype control: Staining with mouse IgG1k-Alexa Fluor 647 and mouse IgG1k eFluor450. Indicated are the numbers of acquired events from both preparations. Presented are data from one of two experiments.

The presence of HIV Env on EVs released by HIV-infected cells was confirmed with another antibody that recognizes gp120, PG16. This glycan-dependent antibody binds to the V2 apex and preferentially recognizes quaternary epitopes that are trimeric^21^. We performed experiments similar to those described above, using MNPs coupled to PG16 to capture particles from the viral preparation. These MNPs capture both HIV and EVs that have been further distinguished from one another by staining for AChE and for CD45. The results of these experiments are presented in Fig. 4 in which the events positive for AChE and/or CD45 represent EVs that carry gp120, through which they have been captured. On average 27.7 ± 1.4% and 72.3 ± 1.4% of EVs captured with PG16-MNPs were positive for CD45 and AChE, respectively. The majority of CD45+ EVs also carried AChE so that 25 ± 0.2% of EVs were double positive for CD45 and AChE (Fig. 4).

**Figure 5:**
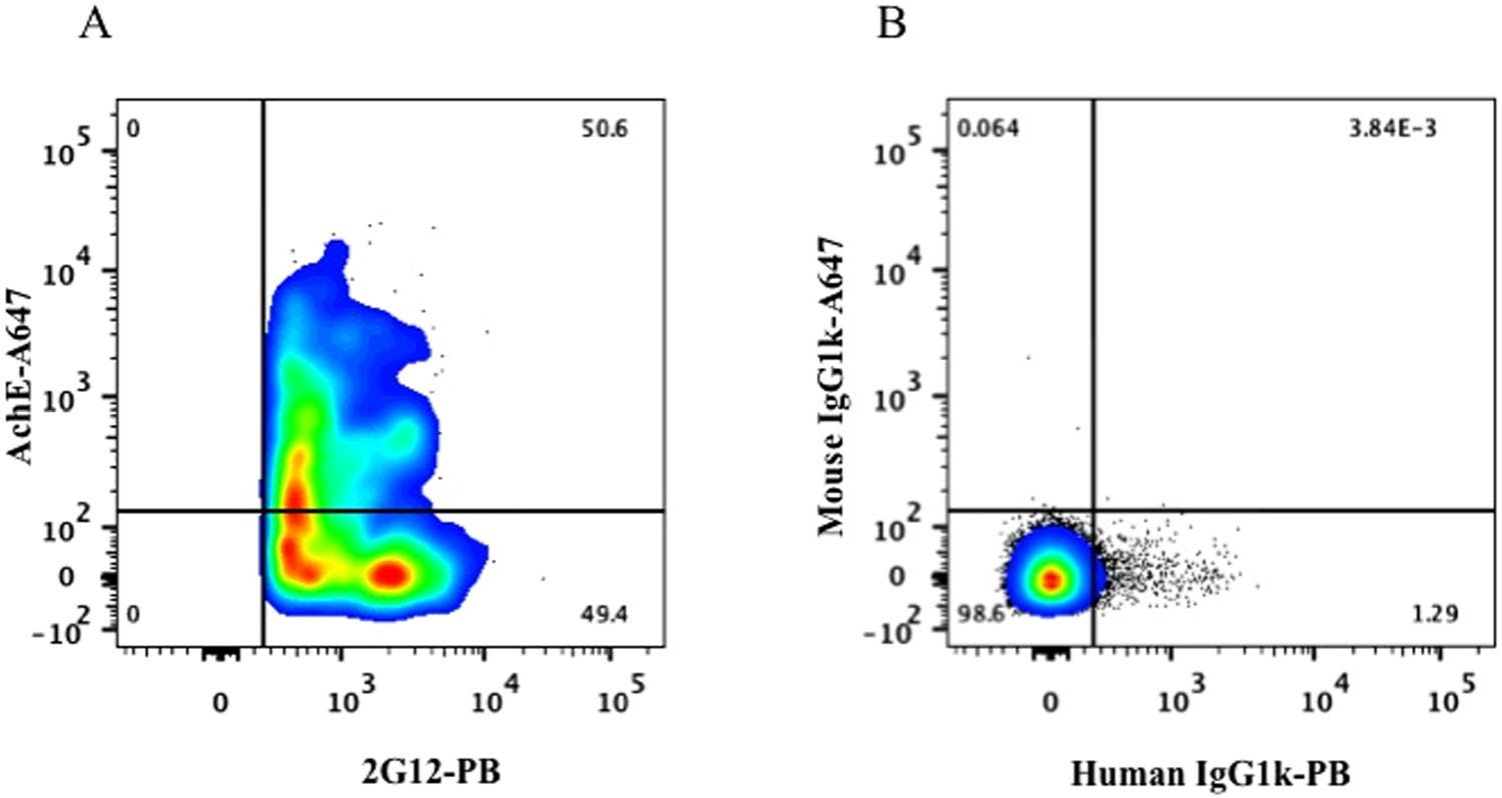
Analysis of EVs expressing gp120 in HIV_LAI.04_ preparation. (**A**) A preparation of HIV_LAI.04_ (containing both viruses and EVs) was incubated with CD81-MNPs and the captured particles were stained with anti- AChE-Alexa Fluor 647 and anti-gp120 2G12-Pacific Blue antibodies. (**B**) Isotype control: Staining with mouse IgG1k-Alexa Fluor 647 and human IgG1k-Pacific Blue. Presented are data from one representative experiment out of three.

The results presented above were obtained with HIV-1 isolate SF162, a prototypical CCR5-tropic HIV. To investigate whether cells infected with other HIV isolates release gp120-carrying EVs we used another HIV-1 isolate, a prototypic CXCR4-tropic virus, HIV_LAI.04_. Again, we captured both virions and EVs with anti-CD81-MNPs, visualized gp120 by staining the complexes with 2G12 antibodies, and distinguished EVs from virions by staining for AChE. The results of the analysis of EVs in HIV_LAI.04_ preparations were similar to those in HIV_SF162_ preparations: 54 ± 0.5% (n = 3) of 2G12-positive events were also positive for AChE, thus representing EVs (Fig. 5).

In summary, a significant number of EVs released by HIV-1 infected cells carry HIV Env protein gp120.

### Depletion of HIV preparation of EVs decreases viral infection of human lymphoid tissue *ex vivo*

To evaluate the effects of EVs on HIV infection, we compared tissue infection by the original viral preparation and the preparation depleted of specific EVs, in particular of gp120+ EVs. In these experiments, we depleted HIV_SF162_ preparations using MNPs coupled to anti-CD45 and to anti-gp120 antibodies PG16 and 2G12. As controls we mock-depleted viral preparations with MNPs coupled to mouse IgG1κ (isotype control) antibodies. After incubation of the viral preparation with MNPs the captured particles were separated on magnetic columns and the fraction that was not retained (“flow through fraction”) was used for infection of human lymphoid tissue *ex vivo* ^17^. As described earlier^17^, the absolute amounts of HIV produced by infected tissues were donor dependent. In the current series of infections, tissues released over 16 days post inoculation between 10.2 and 57 ng of p24. In the experiments described below we compared infection and controls in matched donor tissues and pooled the data by expressing them as percent of control.

**Figure 6:**
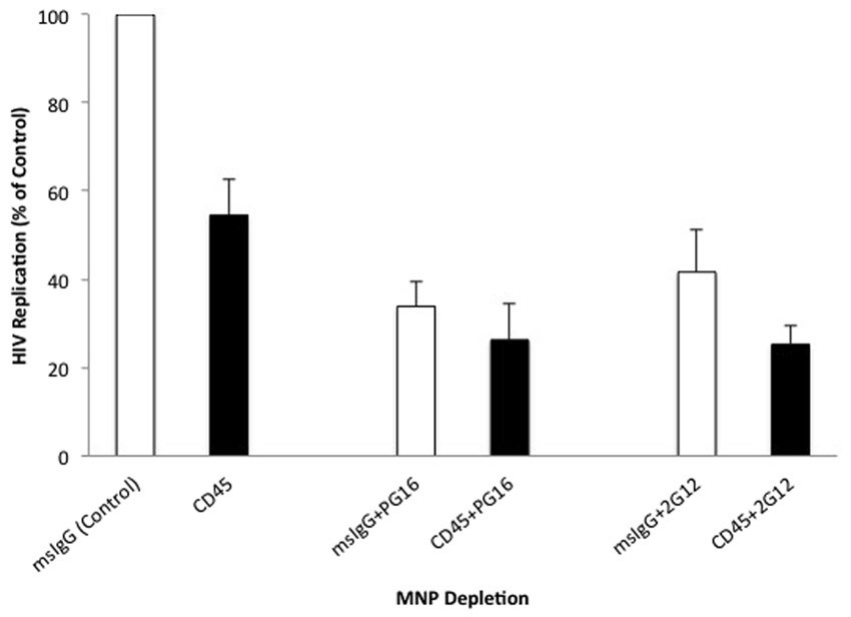
Infection of human tonsillar tissue *ex vivo* with EV-depleted preparations. Donor-matched human tonsillar tissue blocks were inoculated with HIV_SF162_ preparations that underwent one or two rounds of depletion using MNPs coupled to antibodies. Left two bars: Viral preparations were depleted with msIgG-MNPs (“mock-depleted”, control, open bar) or anti-CD45 antibody coupled MNPs (black bar). In the two-round depletion experiments the viral preparations were depleted with MNPs coupled to either 2G12 or to PG16 and next depleted with either MNPs coupled to msIgG (“mock-depleted”, open bars) or to anti-CD45 (black bars). Virus released in the medium over 16 days of infection from 27 tissue blocks for each donor was evaluated with p24 measurement and expressed as percent of control. Presented are means ± standard errors of the means (SEM) from experiments with tissues from four donors.

Infection of human tonsillar tissue inoculated with viral suspensions depleted of CD45 EVs was significantly lower than in mock control: 54.5 ± 8.0% (n = 4, p = 0.03) compared with mouse IgG (“msIgG”)-depleted preparations (Fig. 6). This decrease was not due to the depletion of HIV by CD45-MNPs since the loss of p24 by this procedure was negligible and not statistically different from that of mock depletion with the isotype msIgG-MNPs (on average, 1.7 ± 0.1% vs. 3.5 ± 0.6%, n = 3, p = 0.10).

This result was confirmed by evaluating the amount of HIV-1 genomic RNA associated with the CD45 EVs by real-time PCR. The depletion of the viral preparation by CD45-MNPs removed 1.6 ± 0.1% of HIV gag RNA. This was similar to the amount of HIV genomic RNA removed by isotype msIgG-MNPs (2.4 ± 0.1%).

To evaluate whether this effect was mediated by all CD45-positive EVs or predominantly by those that carry Env, we first depleted the viral preparation of particles carrying Env by using MNPs coupled either to 2G12 or PG16 antibodies and then additionally depleted CD45-positive EVs using MNPs coupled to anti-CD45 antibodies. Controls were depleted with MNPs coupled either to 2G12 or PG16 antibodies and then additionally mock-depleted using MNPs coupled to isotype mouse IgG antibodies. As shown in Fig. 6, depletion of the viral preparation of particles (virions and EVs) that are recognized by anti-gp120 antibodies decreased the tissue infection to the level of 41.6 ± 6.1% (n = 3, p = 0.03) in the case of PG16 and to 43.8 ± 7.5% (n = 4, p = 0.003) in the case of 2G12 as compared to msIgG control depletion. An additional depletion of these preparations of CD45-positive EVs did not result in a significant (p > 0.2) additional decrease of infection. (Infection was decreased by 26.3 ± 8.1% (n = 4) in the case of PG16 plus CD45 depletion and 25.3 ± 4.3% (n = 3) in the case of 2G12 plus CD45 depletion compared to PG16 or 2G12 plus msIgG depletion).

## Discussion

Viral Env (gp120) is a protein that mediates HIV-1 binding and fusion to target cells. However, the role of gp120 is not limited to these functions. Also, it facilitates HIV-1 infection in indirect ways: it has been shown that gp120 interaction with cell surface activates cells^22^ making them preferential targets for HIV infection. Direct interaction of HIV-1 gp120 with pDCs interferes with TLR9 activation resulting in a decreased ability of pDCs to secrete antiviral and inflammatory factors^23^. Most recently it was found that gp120 interaction with the cellular integrin α_4_β_7_ facilitates HIV-1 infection^24, 25^, while blocking these interactions prevents HIV transmission and dissemination^26^. Here, we found that not only virions carry gp120, but these molecules are also incorporated in extracellular vesicles that are released by HIV-infected cells and, as with virions, gp120 in EVs may facilitate viral infection.

In general, vesicles are generated by many, if not all cells of the organism and constitute a highly diverse population of lipoprotein membranes that enclose aqueous space and may contain various biological molecules^1, 27, 28^. Biogenesis of these EVs inside the cells resembles that of retroviruses, in particular of HIV, and as a result, EVs share with these viruses many chemical and physical properties^3^. It is now understood that since EVs are released by HIV-infected cells, any HIV preparation is in fact a mixture of virions and EVs.

In the present work we focused on two questions regarding EVs released by HIV-infected cells: (i) whether these EVs carry viral Envs, and (ii) whether EVs affect HIV infection.

EVs are very diverse in their composition and cargo and may reflect the composition of the cells that released them. In spite of their diversity, until recently EVs were analyzed in bulk, where individual characteristics of each vesicle are lost. Here, in the present work, we used our recently developed flow technology that allows analysis of the antigenic composition of individual EVs stained with fluorescent antibodies^29, 30^. Since EVs are too small to trigger light scattering, which is used in contemporary flow cytometers, we used fluorescence triggering. However, in this case, fluorescent events that correspond to EVs were difficult to distinguish from free antibodies and their aggregates. To overcome these problems we captured EVs with 15-nm magnetic particles (MNPs) coupled with antibodies against one of the EVs’ surface antigens and separated them from unbound EVs and free antibodies in a high magnetic field. We showed that this technique is highly efficient as in the first round of isolation more than 99% of particles that carry the antigen of interest are captured and can be analyzed^30^. Furthermore, we demonstrated that more than 95% of analyzed events represent individual particles, virions or EVs^16, 30^. Capture of the particles with MNPs coupled to specific antibodies and staining the captured particles for other antigens can help to distinguish virions from EVs provided that there are antigens that are specific for only one of these entities.

In general, because of the similarity in biogenesis, the majority of cellular proteins that are incorporated into released particles are shared by EVs and HIV-1^4^. These include tetraspanins (CD81, CD63) that are widely used as markers of EVs in uninfected systems, as well as several other proteins including Alix, TSG101, MHC II^31, 32^, etc. Fortunately, there are at least two cellular proteins that are incorporated in EVs but not in HIV virions: these are CD45^14^ and AChE^15^.

Using the tetraspanin CD81 that is shared by EVs and HIV, we captured both HIV virions and EVs with MNPs coupled with anti-CD81 antibodies and then identified EVs by the presence of either CD45 or AChE. Surprisingly, when we stained our preparation with fluorescent anti-Env (anti-gp120) 2G12 antibodies, approximately 50% of the events were positive both for EVs’ markers and for gp120. The results were similar whether we used CD45 or AChE for identification of EVs, whether we used 2G12 or PG16 as anti-Env antibodies, and whether we used prototypical CXCR4 or CCR5 viral preparations.

In these experiments, our analysis was restricted to EVs that carry CD81 and either CD45 or AChE and thus readily distinguishable from virions. It is reasonable to assume that there are other EVs, CD45-negative and AChE-negative, that carry gp120 and may affect viral infection. Earlier it was reported that EVs that carry Nef ^9, 33^, gag^34^ and trans activation elements^35^ as well as cellular proteins^36^ affect HIV infection.

Env-carrying EVs identified in the present analysis can be classified either as EVs or as defective (non-infectious) viruses and can be part of the population of defective HIV that is known to constitute more than 95% of HIV virions *in vivo* ^37^. In general, Env-carrying EVs seem to be similar to specific fractions of defective HIV-1 virions since CD45- and AChE-positive EVs carrying gp120 do not carry a significant amount of p24.

Do EVs, which seem to be a part of HIV preparations, affect viral infection? We addressed this question by inoculating human lymphoid tissue *ex vivo* with viral suspension depleted of specific EVs. Unfortunately, obvious experiments with two pure fractions of individual HIV virions and individual EVs, mixed in different proportions and tested for infectivity are not feasible in part because there are no reliable methods for total separation of these entities (at least, the viral fractions seem to be contaminated with EVs), and moreover, as mentioned above, the difference between non-infectious virions and Env-positive EVs is purely semantic.

Nevertheless, we attempted to evaluate the effect of EVs on HIV infection by depleting viral preparations of EVs. We used MNPs coupled to specific antibodies to deplete HIV_SF162_ preparations of CD45-positive EVs. Earlier, we showed that capture of particles with MNPs coupled to specific antibodies is highly efficient and less than 1% of the EVs of interest may remain in the depleted preparation^30^. With this CD45-negative preparation we inoculated human lymphoid tissue *ex vivo*. The system of lymphoid tissue *ex vivo* has advantages over isolated cell cultures since it more faithfully reflects lymphoid tissue *in vivo* where critical events of HIV pathogenesis occur. In particular, this system retains its cytoarchitecture and does not require artificial stimulation or activation for productive HIV infection.

Depletion of the HIV_SF162_ preparation of CD45+ EVs decreased infection of human tonsillar tissue by approximately 50%. This decrease occurred because of EVs depletion rather than concomitant depletion of viruses. Indeed the amount of p24 or of HIV genomic RNA depleted by CD45-MNPs was negligible and was not different from the depletion of p24 or HIV RNA by isotype control MNPs that also served as a control for tissue infection. The negligible amount of HIV genomic RNA associated with CD45+ EVs is in agreement with the current view that EVs do not package genomic HIV RNA.

Obviously when we depleted viral preparations with CD45-MNPs we capture both EVs that contain gp120 and those that are gp120-negative. In an attempt to understand which EVs are more important for viral infectivity, we used two antibodies to deplete viral preparations of gp120 containing particles. We depleted HIV preparations with 2G12-MNPs or with PG16-MNPs. In both cases we depleted fractions of HIV-1 together with gp120-positive EVs that are recognized by these antibodies. As expected, the infection of human lymphoid tissue by these depleted fractions was lower than in controls mock-depleted by mouse IgG isotype MNPs.

Next, we additionally depleted these preparations (already depleted of gp120 positive particles) with CD45-MNPs or with control mouse IgG-MNPs and again tested these preparations for infectivity. When the preparations depleted by 2G12- or PG16-MNPs were additionally depleted with CD45-MNPs, we observed only mild additional decrease of infection. This indicates that the decrease of infectivity of the initial preparation when we depleted it of total CD45 EVs was due predominantly to the depletion of Env-containing CD45-positive EVs. Thus, our results indicate that EVs, in particular the ones carrying gp120 may be a positive factor in HIV infection. The effects of EVs on infection may not be similar with all HIV-1 isolates in all systems and may depend on the particular types and quantities of released EVs. Also, CD45 or AChE –negative EVs not identified in the present work may affect HIV infection as well.

The earlier reported effects of EVs on viral infection seem to be multifactorial and to include positive and negative influences probably depending on EVs’ composition^9, 33, 36^. EVs that carry gp120 may facilitate HIV infection by mechanisms similar to those described for gp120 itself ^22^. A similarly facilitating role for EVs was recently described for tumor metastasis when EVs prime specific tissue sites for adhesion of tumor cells^38^.

In summary, our work demonstrates that HIV-infected cells release not only virions but also EVs and that some EVs carry viral Env making them indistinguishable not only physically but also semantically from virions, in particular from those that are defective and are not capable of replication. However these EVs that carry Env identified here facilitate HIV infection and thus may constitute a new target for anti-viral strategy.

## Materials and Methods

### Viral preparations

CCR5-tropic HIV-1_SF162_ (37.8 ng of p24/ml) and CXCR4-tropic HIV-1_LAI.04_ (57 ng of p24/ml) (not depleted of CD45) were obtained from VQA (Virology Quality Assurance Laboratory, Rush University, Chicago, IL).

### Capture and detection of HIV virions and EVs

Viral particles/EVs were captured from viral preparations via magnetic nanoparticles (MNPs) (Ocean NanoTech, Springdale, AR) coupled to anti-CD81 (TAPA-1)(Biolegend, San Diego, California), anti-CD45 (HI30) (Biolegend) or anti-gp120 (Polymun Scientific, Austria) monoclonal antibodies (Abs) as previously described^16, 30^. After coupling, antibody-MNP complexes were suspended in 2 ml of wash/storage buffer and stored at 4 °C. In order to visualize antibody-MNP complexes, we incubated the complexes with Zenon Alexa Fluor 488 or Zenon Alexa Fluor 350 Fab fragments for 30 min at room temperature, then washed them twice with PBS on 100 K Nanosep® (Pall Corporation, Port Washington, NY) centrifugal devices and re-suspended them in the initial volume. For capturing HIV/EVs, labeled Ab-MNP complexes (~3.9 × 10^12^ particles in 60 µl) were incubated with HIV viral preparation (~8 × 10^6^ virions in 60 µl) at 37 °C for 40 minutes with continuous mixing. Next, different combinations of fluorescent detection antibodies (anti-AChE-Alexa Fluor 647 (Merck Millipore, Billerica, MA), anti-CD45-eFluor450 (BD Bioscience, San Jose, CA), 2G12-Alexa Fluor 647 were added to the mixture and incubated at room temperature for 20 minutes. Control preparations were stained with isotype antibodies. The captured and stained complexes were then separated from unbound HIV/EVs and unbound fluorescent antibodies on magnetic columns (Miltenyi Biotech, San Diego, CA) in a high magnetic field generated by an OctoMACS magnet (Miltenyi Biotech), washed three times with 500 µl of washing buffer (0.5%BSA, 2 mM EDTA in PBS) and eluted with 400 µl PBS from the column removed from the magnet and fixed with 1.5% paraformaldehyde.

### Analysis of HIV virions with flow cytometer

We analyzed purified complexes on an LSRII (BD Biosciences) flow cytometer equipped with 355-, 407-, 488-, 532- and 638-nm lasers by triggering on fluorescence. The background level of fluorescence was evaluated with 0.1-µm filtered PBS and the threshold was set to the lowest fluorescence channel that did not generate an AlexaFluor 488/AlexaFluor 350 signal with this solution. The detailed gating strategy was described in ref. 16. Data were acquired with Diva 6.3 and were analyzed with FlowJo software v9.4.9 (Treestar Software, Ashburn, OR).

### Depletion of HIV viral preparations

MNPs (~6 × 10^12^ particles in 100 µl) coupled to mouse IgG (used as control), anti-CD45, anti-PG16 or anti-2G12 antibodies were added to viral preparations of HIV_SF162_ (~5 × 10^7^ virions in 400 µl) and incubated for 2 hours at 4 °C. Captured HIV/EVs were separated on magnetic columns, while the non-captured fractions were collected and used for further infection of tonsillar tissue *ex vivo*.

### Human *ex vivo* tissues

Human tonsillar tissues were obtained from routine tonsillectomy (unrelated to the current study) performed in the Children’s Hospital (Washington, DC). Tissues were received from the Pathology Department and were considered as “pathological waste”. Tissue samples were anonymized and the protocol was approved by the NIH Office of Human Subject Research. Human tonsillar tissues were dissected into small blocks with 27 blocks per condition, as previously described^17^ and inoculated with viral preparations depleted with MNPs coupled with anti-mouse IgG, CD45, PG16 or 2G12 Abs. Tissues were cultured for 16 days with medium being collected and changed every 3 days.

Replication of HIV was evaluated by measurement of p24 released into culture medium using dynamic immunofluorescent cytometric bead assay^39^.

### Quantification of HIV-1 genomic RNA

Quantification of HIV-1 genomic RNA was performed as previously described^16^. Briefly, 100 µl aliquots of both the MNP-virus complexes eluted from magnetic column and the flow through fraction were subjected to nucleic acid extraction on NucliSENS Biomerieux EasyMag 2.0 instrument (BioMerieux, Durham, NC). One step real-time PCR was performed with qScript One-Step Fast MGB qRT-PCR kit (Quanta Biosciences, Gaithersburg, MD) using the following primer set: HIV-1 gag F1: GAGGCTAGAAGGAGAGAGATGGGT, HIV-1 gag R1: CCCTGGCCTTAACCGAATTT and the SR73 probe: VIC-GGG TGC GAG AGC GTC AGT ATTA-MGB. Amplifications were carried out on a BioRad CFX96 Touch Thermocycler (Hercules, CA) according to the following cycling parameters: 7:30 min at 48 °C, 30 sec at 95 °C followed by 45 cycles of 3 sec at 95 °C and 25 sec at 60 °C.

### Statistical analysis

We conducted statistical analysis using JMP10 (SAS Institute). Results are represented as means ± standard errors of the mean (SEM). The statistical significance of differences between various experimental groups was evaluated with paired Student’s *t* test. All hypothesis tests were two-tailed and a *p* value of ≤0.05 defined statistical significance.
